# Decitabine, a DNA-demethylating agent, promotes differentiation via NOTCH1 signaling and alters immune-related pathways in muscle-invasive bladder cancer

**DOI:** 10.1038/s41419-017-0024-5

**Published:** 2017-12-14

**Authors:** Swathi Ramakrishnan, Qiang Hu, Nithya Krishnan, Dan Wang, Evelyn Smit, Victoria Granger, Monika Rak, Kristopher Attwood, Candace Johnson, Carl Morrison, Roberto Pili, Gurkamal Chatta, Khurshid Guru, Geraldine Gueron, Lacey McNally, Jianmin Wang, Anna Woloszynska-Read

**Affiliations:** 1 Department of Pharmacology and Therapeutics, Roswell Park Cancer Institute, Buffalo, NY 14263 USA; 2 Department of Bioinformatics and BioStatistics, Roswell Park Cancer Institute, Buffalo, NY 14263 USA; 3 Department of Cell Biology, Faculty of Biochemistry, Biophysics and Biotechnology, Jagiellonian University, Kraków 31-007, Poland; 4 Department of Medicine, Indiana University, Bloomington, IN 47405 USA; 5 Department of Medicine, Roswell Park Cancer Institute, Buffalo, NY 14263 USA; 6 Department of Biological Chemistry, School of Sciences, University of Buenos Aires, IQUIBICEN-CONICET, Intendente Guiraldes 2160, CABA, Buenos Aires 1428, Argentina; 7 Department of Medicine, University of Louisville, Louisville, KY 40292 USA

## Abstract

Aberrant DNA methylation observed in cancer can provide survival benefits to cells by silencing genes essential for anti-tumor activity. DNA-demethylating agents such as Decitabine (DAC)/Azacitidine (AZA) activate otherwise silenced tumor suppressor genes, alter immune response and epigenetically reprogram tumor cells. In this study, we show that non-cytotoxic nanomolar DAC concentrations modify the bladder cancer transcriptome to activate NOTCH1 at the mRNA and protein level, increase double-stranded RNA sensors and CK5-dependent differentiation. Importantly, DAC treatment increases ICN1 expression (the active intracellular domain of NOTCH1) significantly inhibiting cell proliferation and causing changes in cell size inducing morphological alterations reminiscent of senescence. These changes were not associated with β-galactosidase activity or increased p16 levels, but instead were associated with substantial IL-6 release. Increased IL-6 release was observed in both DAC-treated and ICN1 overexpressing cells as compared to control cells. Exogenous IL-6 expression was associated with a similar enlarged cell morphology that was rescued by the addition of a monoclonal antibody against IL-6. Treatment with DAC, overexpression with ICN1 or addition of exogenous IL-6 showed CK5 reduction, a surrogate marker of differentiation. Overall this study suggests that in MIBC cells, DNA hypomethylation increases NOTCH1 expression and IL-6 release to induce CK5-related differentiation.

## Introduction

The five-year survival of patients with invasive bladder cancer who present with locally advanced or metastatic disease is less than 25%^1^. Neoadjuvant cisplatin-based chemotherapy (CBC) followed by radical cystectomy remains the first line treatment for muscle-invasive bladder cancer (MIBC) patients over the last three decades. Although CBC is associated with a survival advantage, inherent and acquired cisplatin resistance is frequently observed^1^ and associated with survival rates of <2 years^2^.

Agents targeting DNA methylation such as 5-aza-2’-deoxycytidine (Decitabine, DAC) and 5-azacytidine (Azacytidine, AZA) are FDA-approved for the treatment of myelodysplastic syndrome^3^. These agents, in part, decrease DNA hypermethylation of CG-rich regions (CpG islands) in promoters of tumor suppressor genes and restore transcriptional activity of those loci^4,5^. DNA-demethylating agents (1) induce immune responses^6,7^, (2) reprogram tumors by targeting self-renewing cell population^8,9^ and (3) sensitize tumors to chemotherapy or immunotherapy based upon checkpoint inhibition^6,10^. Targeting DNA methylation in tumors presents a unique opportunity to alter cell transcriptional programs, activate tumor suppressors and immune system regulating genes to achieve therapeutic benefit, either alone or in combination with other anticancer therapies.

NOTCH1 expression can be lost through non-sense mutations in MIBC tumors^11^. We hypothesized that NOTCH1 expression is also lost due to DNA hypermethylation of its promoter region and subsequent transcriptional repression. Mice with an inactive NOTCH pathway have a greater incidence of carcinogen-induced bladder tumor with squamous features and reduced overall survival^12^. NOTCH1 expression and its downstream targets JAGGED-1 and HES-1 are also lost in aggressive forms of MIBC^13,14^. NOTCH1 activation sensitizes osteosarcoma cells to cisplatin treatment^15^. One potential downstream target of NOTCH1 signaling is IL-6, a pro-inflammatory cytokine associated with poor prognosis in patients with different types of solid tumors through activation of the JAK/STAT pathway^16,17^. NOTCH1 has been shown to locate to the IL-6 promoter to increase its expression^18^. IL-6 release in the context of DNA damage-induced senescence is considered to be pro-tumorigenic^19,20^. However in bladder cancer, IL-6 overexpression reduces motility and invasion *in vitro* in MIBC cells with IL-6 knockdown increasing tumor burden *in vivo* ^21^. Our study shows for the first time that DAC restores NOTCH1 expression and promotes IL-6 mediated CK5-differentiation in muscle-invasive bladder cancer cells.

## Results

### Non-cytotoxic decitabine concentrations induce DNA hypomethylation of the NOTCH1 promoter leading to NOTCH1 transcriptional activation

**Fig. 1:**
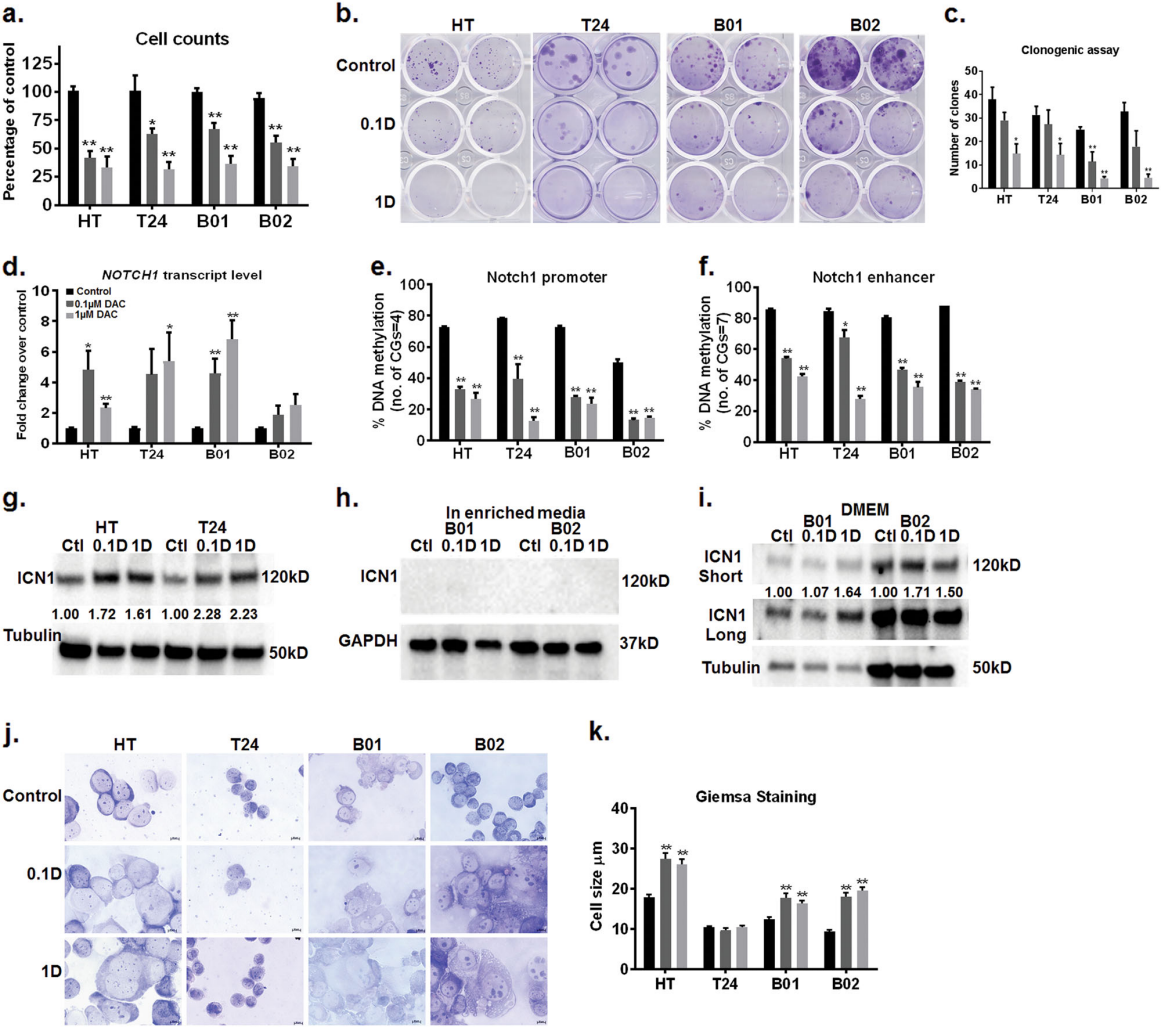
DNA hypomethylation increases NOTCH1 expression and alters cell size Bladder tumor cells were treated with DAC every 48 h for up to 5 days. **a** 0.1 and 1 µM DAC caused a reduction in cell proliferation in all four bladder tumor cell lines. The *Y* axis represents percentage changes in cell counts in DAC-treated cells as compared to control cells. Error bars indicate standard error of means from triplicate experiments and technical duplicates in each experiment. **b**, **c** DAC treatment reduced the number of clones in bladder tumor cells lines. The number of clones were counted and compared to control cells. The *Y* axis represents the number of clones in control and treatment conditions. Error bars indicate standard error of means from triplicate experiments and technical duplicates. **d** DAC induced *NOTCH1* transcript in bladder tumor cell lines. The *Y* axis represents fold change in DAC-treated cells as compared to control cells. Error bars indicate standard error of means from triplicate experiments and technical duplicates in each experiment. **e**, **f** DAC treatment decreased DNA methylation at the NOTCH1 promoter and enhancer regions. The Y-axis represents percentage DNA methylation with error bars indicating standard error of means from duplicate experiments and technical duplicates in each experiment. **g**, **h** Western blot analysis of DAC-treated bladder tumor cells showed an increase in the active intracellular domain of NOTCH1 (ICN1) in HT1376 and T24 but not in B01 and B02. GAPDH was used as the loading control. The western blot is a representative of triplicate experiments. **i** Western blot analysis of B01 and B02 cells cultured in DMEM showed an increase in levels of the active intracellular domain of NOTCH1, ICN1. GAPDH was used as the loading control. The western blot is a representative of triplicate experiments. **j** Bright field images of Giemsa staining showed morphological changes in DAC-treated cells. DAC-treated cells appeared enlarged and flattened as compared to control cells on Day 5. The images are representative of duplicate experiments. **________** represents 10 µm. **k** The horizontal length of 50 cells from each condition was measured by ImageJ. The graph represents an average measurement of 50 cells in control, and DAC-treated groups. The DAC-treated cells were significantly larger as compared to the control cells. Student’s *t*-test was used to compare control and treated cells in all the panels. *p < 0.05, **p < 0.01.

DAC is used for treatment in patients with acute myelocytic leukemia and myelodysplastic syndrome. In these patients, standard DAC therapy generally results in plasma levels in the range of 50nM–1 µM when measured 1–2 h after receiving DAC (15–20 mg/m^2^ of I.V infusion)^22,23^. Because DAC is given intravenously, plasma concentrations are not representative of those in solid tissues. For patients receiving DAC for the treatment of solid tumors, the actual levels which reach the target tumor tissue are likely to be significantly lower. To mimic concentrations that are therapeutically relevant, in our *in vitro* experiments, we used 0.1 and 1 µM DAC. We analyzed: (1) HT1376 and T24 cell lines of epithelial origin with p53 inactivating mutations and (2) B01 and B02^24,25^ patient-derived xenograft cells with squamous differentiation and wild-type p53. Both 0.1 and 1 µM DAC successfully depleted DNA methyltransferase 1 (DNMT1) (Supplementary Fig. 1a), and significantly reduced LINE-1 methylation by 10–20% in T24 and B02 cells (Supplementary Fig. 1b). Although LINE-1 methylation was closer to 10% or less in untreated HT1376 and B01 cells, DAC treatment reduced LINE-1 methylation levels by 2% in these cells (Supplementary Fig. 1b). These results confirm that low nanomolar DAC doses are active in all the cell lines tested. Both 0.1 and 1 µM DAC significantly reduced cell proliferation by greater than 50% without affecting viability in more than 20% of the cells compared to the vehicle (Fig. 1a, Supplementary Fig. 1c). DAC also lowered the ability of cells to form individual subclones compared to control cells (Fig. 1b, c). To delineate the transcriptional mechanisms by which non-cytotoxic DAC doses reduced cell proliferation we used paired-end RNA-sequencing. We used DAC-treated T24 cells with significant decrease in LINE-1 methylation (Supplementary Fig. 1b) for these analyses. RNA sequencing revealed that 166 genes and 350 genes were upregulated in 0.1 and 1 µM DAC-treated T24 cells, respectively, compared to control cells. Interestingly gene enrichment analysis of differentially expressed genes in DAC-treated cells revealed upregulation of the NOTCH pathway (Supplementary Figure 2a and Supplementary Table 1). We confirmed our RNA sequencing results by qPCR and found more than two-fold increase in the *NOTCH1* transcript in all cell lines (Fig. 1d). The increase in *NOTCH1* transcript was significant in HT1376, T24, and B01 cells with an upward trend observed in B02 cells (Fig. 1d). This led us to investigate whether NOTCH1 signaling is clinically important in bladder cancer. RNA-sequencing and survival data from Roswell Park Cancer Institute (RPCI) MIBC patients showed that *NOTCH1* upregulation (*z*-score threshold of +1.5) correlated with greater overall survival and progression-free survival compared with patients with no alterations in *NOTCH1* expression (Supplementary Fig. 2b). We used the TCGA bladder cancer database to confirm these findings. We found NOTCH1 transcript and protein upregulation (*z*-score threshold of +1.5) in 11–12% of patient samples (Supplementary Fig. 2c). These patients exhibited better overall (OS) and progression-free survival (PFS) compared to patients with no alterations in *NOTCH1* expression (Supplementary Fig. 2c). NOTCH1 was methylated in a subset of bladder tumors in both the RPCI and TCGA cohorts (Supplementary Table 3 and Supplementary Fig. 2d). A subset of tumors with methylation β-values >0.3 corresponded with low *NOTCH1* mRNA levels (*z*-scores <1). Therefore, we investigated whether DNA methylation regulates *NOTCH1* expression or activation in bladder cancer cells. NOTCH1 promoter and enhancer regions are hypermethylated (above 70%) in all four cell lines (Fig. 1e–f). DAC demethylated both the promoter and enhancer regions to below 50%; this correlated with increased *NOTCH1* transcript (Fig. 1e–f). DAC-treated HT1376 and T24 cells have greater ICN1 protein expression, the active transmembrane domain of NOTCH1, compared to control cells (Fig. 1g). These results show for the first time that DNA methylation contributes to the transcriptional regulation of *NOTCH1* expression in bladder cancer cells.

B01 and B02 cells are cultured in media containing cholera toxin which degrades NOTCH1 and ICN1 protein^26^ (Fig. 1h). To confirm this, we used short-term cultures of B01 and B02 in regular DMEM media without cholera toxin. DAC-treated B01 and B02 cells cultured in DMEM had higher ICN1 expression as compared to control cells (Fig. 1i). For the remainder of the study, we used B01 and B02 cells cultured in specialized F12K/DMEM media: (1) to avoid selective pressure on B01 and B02 cells by culturing them in regular DMEM media and (2) to investigate NOTCH independent anti-proliferative mechanisms of DAC treatment.

### DAC and NOTCH1 mediate IL-6 release and morphological changes in MIBC cells

**Fig. 2:**
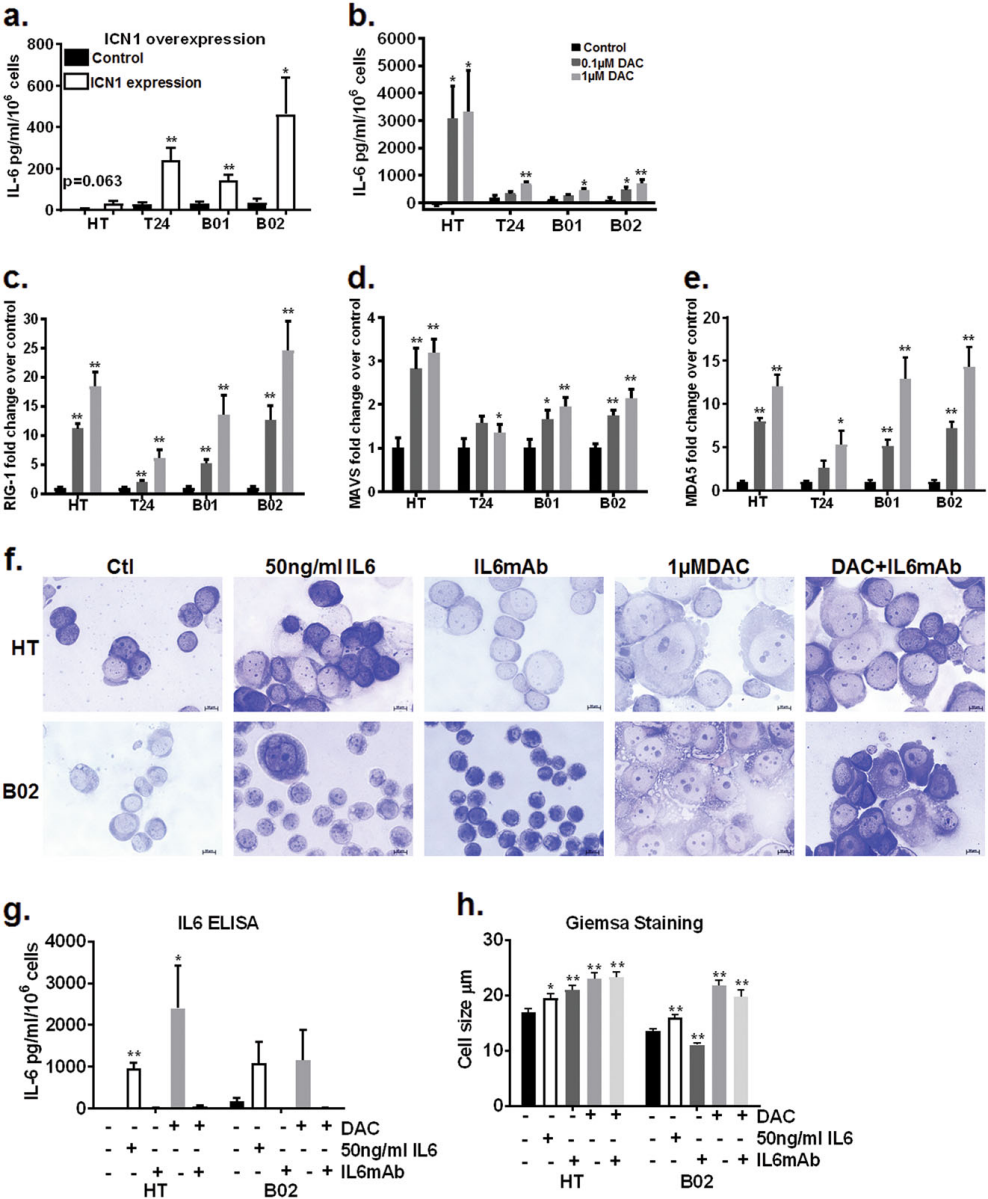
IL-6 is a downstream mediator of DAC-induced morphological alterations in an NOTCH1 dependent and independent manner **a** ICN1 transient overexpression increased IL-6 release in cell supernatants at 72 h. The *Y* axis indicates IL-6 in pg/ml normalized for 1 × 10^6^ cells from triplicate experiments and technical duplicates with error bars indicating standard error of means. **b** Bladder tumor cells were treated with DAC every 48 h for up to 5 days. DAC caused IL-6 release in bladder tumor cell lines with a robust increase in HT1376 cells. The Y-axis indicates IL-6 in pg/ml normalized for 1 × 10^6^ cells from triplicate experiments and technical duplicates with error bars indicating standard error of means. **c–e** mRNA levels of double-stranded RNA sensors increased in DAC-treated cells. The Y-axis indicates average fold change over control cells from triplicate experiments and technical duplicates with error bars indicating standard error of means. Student’s t-test was used to compare control and treated cells. *p < 0.05, **p < 0.01. **f** Bright field images of Giemsa staining showed morphological changes in cells with exogenous IL-6. Cells with exogenous IL-6 appeared enlarged and flattened as compared to control cells. The addition of IL-6 monoclonal antibody reduced the appearance of DAC-induced enlarged cell morphology. _________ represents 10 µm. **g** The addition of a monoclonal antibody against IL-6 with DAC treatment inhibited IL-6 release as compared to DAC treatment alone. The Y-axis indicates IL-6 in pg/ml normalized for 1 × 10^6^ cells from triplicate experiments and technical duplicates with error bars indicating standard error of means. **h** The horizontal length of 50 cells from each condition was measured by ImageJ. The graph represents an average measurement of 50 cells in control and treated groups. Student’s *t*-test was used to compare control and treated cells. *p < 0.05, **p < 0.01.

Giemsa staining revealed that DAC-treated cells underwent morphological changes compared to untreated cells (Fig. 1j, k). Except T24 cells, DAC-treated HT1376, B01, and B02 cells appeared enlarged compared to controls (Fig. 1j, k). We used flow cytometry to confirm this morphological change, where we observed increase in channel number which corresponds to higher voltage and therefore greater cell size in DAC-treated cells compared to control cells (Supplementary Fig. 1d, e). To analyze whether morphological alterations were driven in part by NOTCH1 activation, we transiently overexpressed ICN1 in HT1376, T24 and B01/B02 cells cultured in DMEM. ICN1 overexpression reduced cell proliferation with cells appearing enlarged compared to control cells (Supplementary Fig. 3a, b). These morphological changes were reminiscent of a senescent phenotype^27,28^. Therefore, DAC-treated cells and ICN1 overexpressing cells were tested for β-gal staining and p16 expression to identify if DAC and/or NOTCH1 activation causes senescence. Only a fraction of DAC-treated T24 and B01 cells stained positively for β-gal (Supplementary Fig. 3e). Since β-gal staining was high in untreated HT1376, it was not possible to determine if DAC treatment caused an increase in β-gal staining (Supplementary Fig. 3e). There was no observable increase in β-gal staining in DAC-treated B02 cells (Supplementary Fig. 3e). DAC-induced p16 only in HT1376 cells and was not detectable in the other three cell lines (Supplementary Fig. 3f). 0.1 µM DAC treatment-induced p27 expression in HT1376 and B02 cells (Supplementary Fig. 3g). Although we did not measure c-myc a downstream target of p27^9^, another study has shown that nanomolar DAC concentrations reduce c-myc expression^29^. ICN1 overexpressing cells had a low number of β-gal positive cells and no p16 induction as compared to control cells (Supplementary Fig. 3c, d). IL-6 release in the media was used as an additional measure of senescence-associated secretory phenotype in both DAC-treated and ICN1 overexpressing cells. ICN1 overexpressing cells had higher IL-6 in cell supernatants (greater than 200 pg/ml/10^6^ cells) compared to control cells (Fig. 2a). DAC-treated HT1376 cells also maintained high IL-6 release (greater than 2000pg/ml/10^6^ cells) compared to DAC-treated T24, B01 and B02 cells (Fig. 2b). NOTCH1 is a known upstream regulator of IL-6 signaling^18,30^. It is possible that greater IL-6 release in HT1376 cells was due to DAC-induced ICN1 expression, though IL-6 release was also observed in B01 and B02 cells cultured in enriched media where ICN1 protein expression was not detected (Fig. 2b). IL-6 release can be triggered by the presence of double-stranded RNAs^31,32^. DAC treatment has been shown to upregulate a viral defense pathway through an increase in double-stranded RNAs in colorectal and ovarian cancer cell lines^6,7^. DAC concentrations in the µM range also activate the IFN-γ pathway in p53 mutant mouse embryonic fibroblasts (MEFs)^33^. In bladder cancer cells, DAC treatment increased the double-stranded RNA sensors, *MDA5*, *MAVS* and *RIG-1* (Fig. 2c–e), intermediate mediator *IRF7*, and downstream targets *IFI44* and *IFI27* and *IFN-γ* by 2-fold to 70-fold compared to control cells (Supplementary Fig. 4a–d). HT1376 cells exhibited 10–20 fold induction of downstream targets IFI27 and IFI44 compared to 5-fold to 10-fold change T24, B01 and B02 cells (Supplementary Fig. 4c, d). These data suggest that DAC treatment can also increase IL-6 levels by upregulating double-stranded RNAs. The massive amount of IL-6 release observed with DAC treatment and ICN1 overexpression does not explain and is disproportionate to the small number of β-gal positive cells. Senescence-associated IL-6 secretion is usually associated with DNA damage^19,20^. DAC did not induce γH2A.X associated DNA damage in HT1376, T24 and B01 cell lines but increased γH2A.X expression in B02 cells (Supplementary Fig. 4e, f). The low levels of β-gal positivity (Supplementary Fig. 3c, e), negative p16 expression (Supplementary Fig. 3d, f), p27 induction (Supplementary Fig. 3g) and the increase in γH2A.X observed only in B02 cells (Supplementary Fig. 4e, f) indicate that it is unlikely that IL-6 release is related to senescence in this case.

### IL-6 mediates morphological changes and CK5-related differentiation in DAC-treated bladder cancer cells

**Fig. 3:**
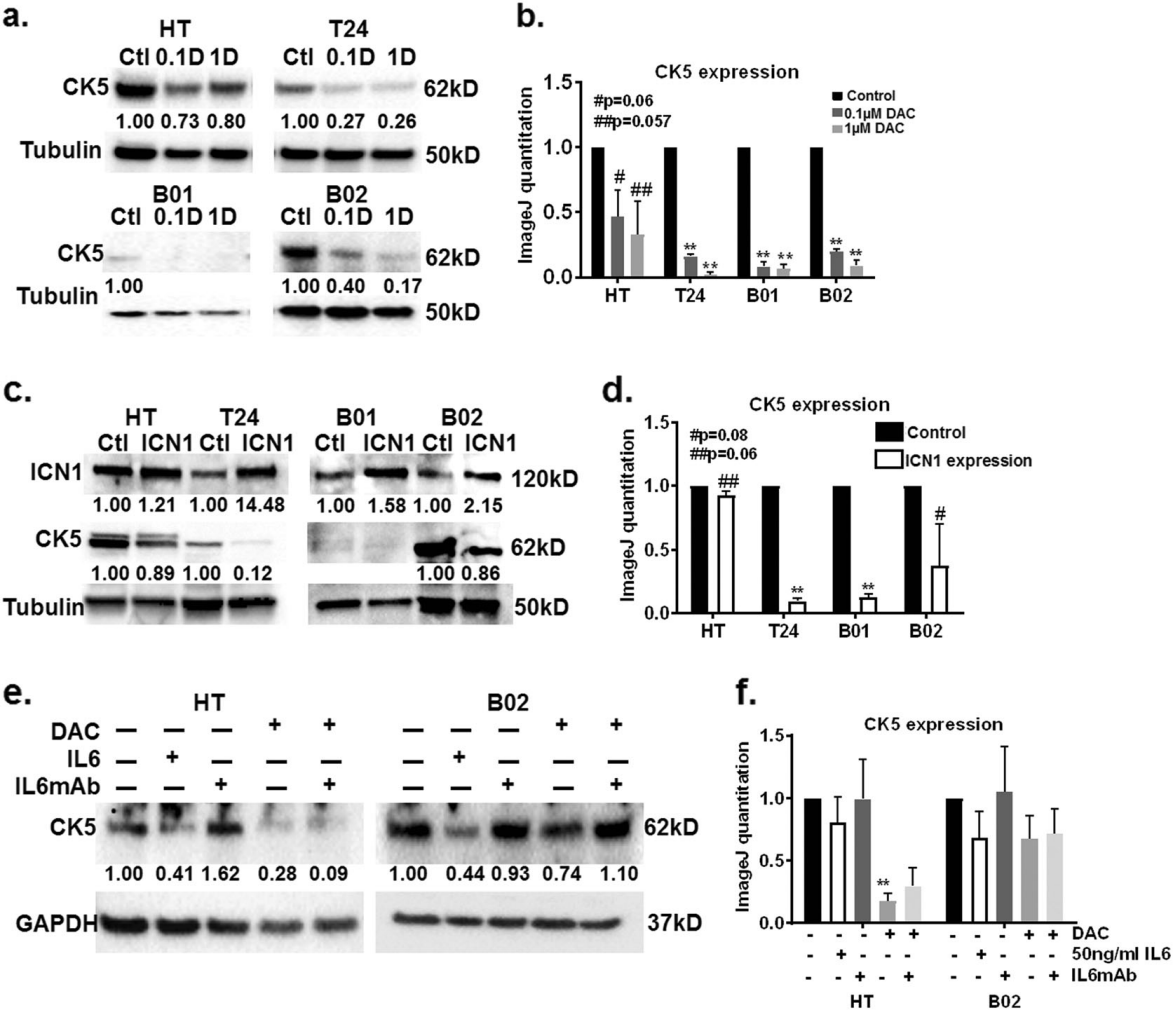
IL-6 and NOTCH1 are mediators of CK5-related differentiation in DAC-treated cells **a** Bladder tumor cells were treated with DAC every 48 h for 5 days. CK5 protein expression is downregulated in DAC-treated cells on day 5. GAPDH was used as loading control. The western blot is a representative of triplicate experiments. **b** Western blots from triplicate experiments were quantitated by ImageJ. CK5 values were normalized to Tubulin from each condition and plotted on the graph. **c** CK5 protein expression is downregulated in ICN1 overexpressing cells. GAPDH was used as loading control. The western blot is a representative of triplicate experiments. **d** Western blots from triplicate experiments were quantitated by ImageJ. CK5 values were normalized to Tubulin from control and ICN1 overexpressing cells and plotted on the graph. **e** The addition of 50ng/ml IL-6 reduced CK5 expression in HT and B02 cells. The addition of a monoclonal antibody against IL-6 rescued CK5 expression only in DAC-treated B02 cells. GAPDH was used as loading control. The western blot is a representative of triplicate experiments. **f** Western blots from triplicate experiments were quantitated by ImageJ. CK5 values were normalized to GAPDH from each condition and plotted on the graph.

To test if DAC-mediated IL-6 release activates downstream IL-6 signaling, we measured phospho-STAT3 levels, a well-established readout of the IL-6 signaling cascade^34^. p-STAT3 and STAT3 protein levels did not increase by greater than two-fold in DAC-treated cells (Supplementary Figure 5a), and p-STAT3 and STAT3 levels were depleted 24 h after DAC addition (Supplementary Figure 5b). Our data suggests that IL-6 release occurs without sustained activation of the JAK/STAT pathway. Therefore, we tested if IL-6 is a mediator of morphological alterations in DAC-treated cells. Exogenous IL-6 addition led to enlarged cell morphology (Fig. 2f, h). Increase in p-STAT3 levels 30 min after IL-6 addition showed that exogenous IL-6 is active in cells (Supplementary Fig. 5c). Exogenous IL-6 and monoclonal antibody against IL-6 did not cause significant change in cell proliferation alone or in combination with DAC (Supplementary Fig. 4g). The addition of a monoclonal antibody against IL-6 in DAC-treated cells reduced the appearance of enlarged cells in comparison to DAC treatment alone (Fig. 2f–h). We hypothesized that morphological alterations are indicative of differentiation^8,35^. CK5 positive basal stem-like cells are thought to be the progenitors of carcinoma in situ, muscle-invasive and squamous cell carcinoma lesions^36^. Therefore, we used the loss of CK5 protein expression as a surrogate marker of differentiation in DAC-treated and ICN1 overexpressing cells. DAC treatment reduced CK5 protein expression suggesting that these cells are losing the population of cells that can increase tumorigenic potential (Fig. 3a, b). Transient ICN1 overexpression downregulated CK5 protein expression (Fig. 3c, d) suggesting that these cells are also undergoing CK5-related differentiation. Exogenous IL-6 reduced CK5 protein levels in HT1376 and B02 cells (Fig. 3e, f). Monoclonal antibody against IL-6 suppressed DAC-mediated IL-6 release and rescued the CK5 expression in DAC-treated HT1376 and B02 cells (Fig. 3e, f). These results suggest that IL-6 is a mediator of CK5-related differentiation in MIBC cells.

### *NOTCH1*, *IL-6* and *IFN-γ* and other related transcripts are altered in muscle-invasive bladder cancer patients

**Fig. 4:**
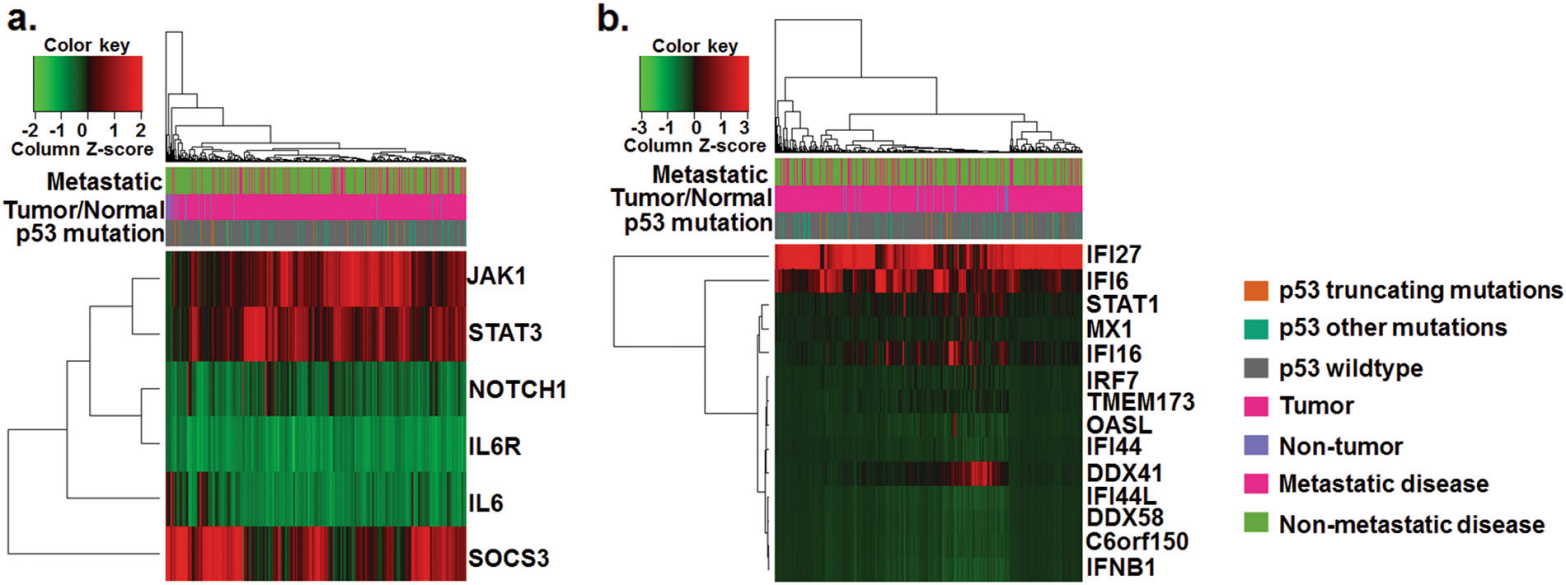
Differentiation-associated transcripts; NOTCH1-IL-6 and viral defense associated transcripts were downregulated in the TCGA bladder tumor cohort Normalized mRNA *z*-scores of TCGA bladder tumor data set revealed downregulation of (**a**) *NOTCH1* and *IL-6* transcripts, and (**b**). Viral defense transcripts. This downregulation occured independently of p53 expression and metastatic disease. Each row is a gene *z*-score and each column is an individual tumor sample. Red color indicates that the gene was expressed at a higher level than the gene with the green color code within the same tumor.

**Fig. 5:**
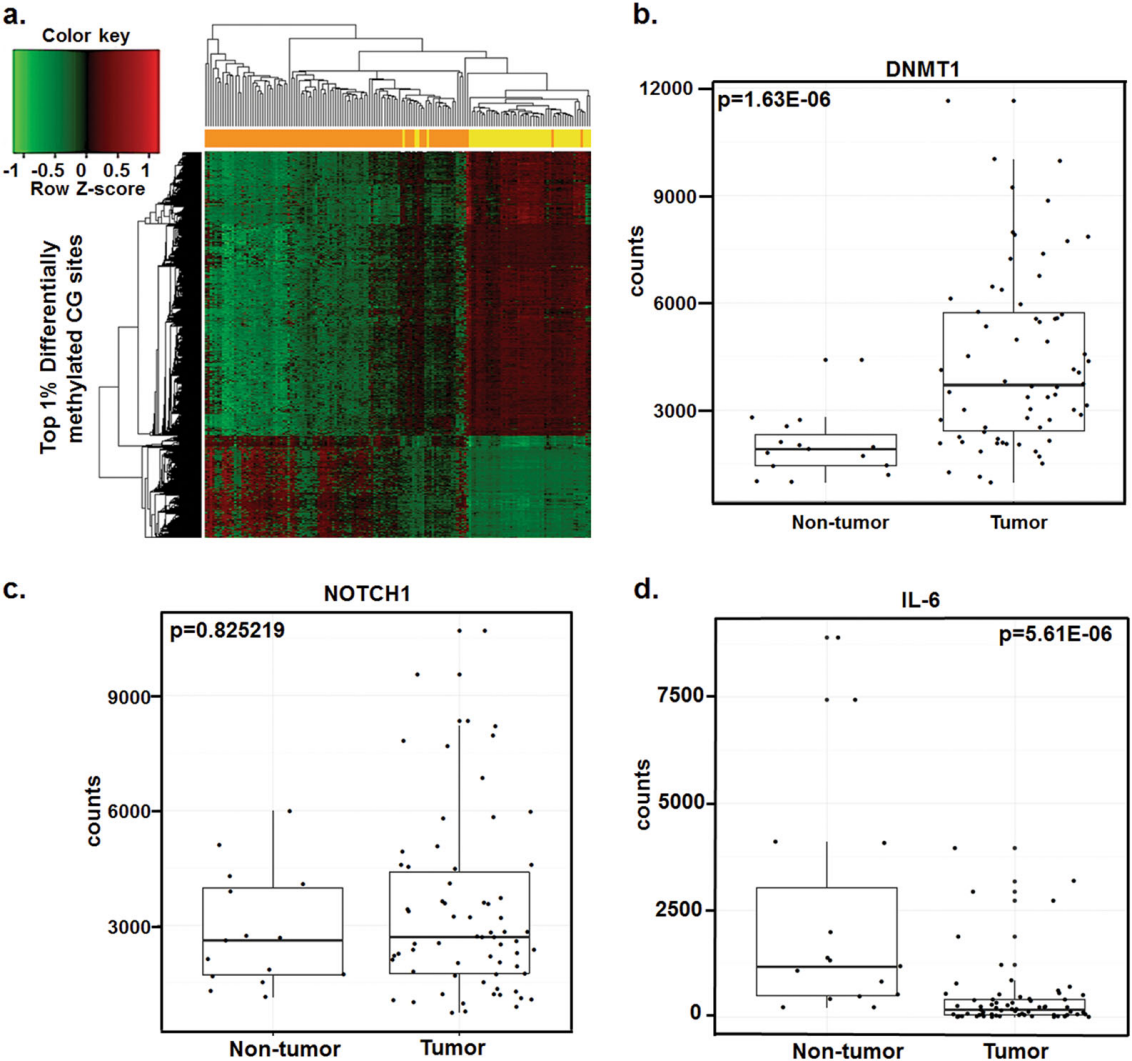
DNA methylation and RNA sequencing data reveal differential methylation and transcript levels in the RPCI cohort **a** DNA methylation of 174 (including 74 matched tumor and non-tumor samples) bladder cancer specimens distinguished between tumor (orange) and non-tumor tissue (yellow). Red color indicates hypermethylation and green color indicates hypomethylation. Each row is a CG site, and each column is a different tumor sample. Top 1% differentially methylated CG sites are represented in the heat-map. **b**–**e** RNA sequencing was performed on 66 bladder tumor specimens (including 15 matched tumor and non-tumor samples). Transcript levels of *DNMT1*, *CDKN1A*, *NOTCH1*, and *IL-6* were significantly lower in tumor tissues as compared to non-tumor tissues.

Our *in vitro* studies show DAC activation of immune-related (IL-6 and viral defense) and differentiation-associated (NOTCH1) transcripts and proteins in bladder cancer cell lines. To evaluate if these are clinically relevant pathways, baseline mRNA levels of these genes and their methylation status were measured in the MIBC patients at Roswell Park Cancer Institute and TCGA data (see materials and methods). *NOTCH1*, *IL-6*, and *IL-6R* mRNA levels were consistently downregulated in the TCGA cohort (Fig. 4a). Downstream IL-6 signaling *JAK1*, *STAT3*, and *SOCS3* were consistently upregulated in the same bladder tumors with low IL-6 levels (Fig. 4a) suggesting IL-6 independent activation of the JAK1/STAT3 pathway. *STAT1* the intermediate signal in viral defense pathway was frequently downregulated in bladder tumors (Fig. 4b). The established signature for the viral defense mechanism^6,7^ was also downregulated in MIBC patient samples (Fig. 4b). In patient samples from RPCI, global DNA methylation alone can differentiate between adjacent non-tumor and tumor bladder tissues (Fig. 5a). Also, DNA methyltransferase mRNA was upregulated in tumors compared to non-tumor tissues (Fig. 5b). Pathway enrichment analysis of differentially methylated genes revealed that NOTCH belongs to one of the top ten hypermethylated networks in MIBC patients in the Roswell Cohort (Supplementary Table 3). Both *NOTCH1* and *IL-6* were downregulated (based on raw counts from RNA sequencing and *z*-scores <0) in a subset of RPCI patients (Fig. 5c, d). Overall, the low levels of NOTCH/IL-6 and immune-related genes indicate profound dysregulation of tumor-suppressive pathways in bladder tumors.

## Discussion

DNA-demethylating agents reduce proliferation in cell lines and xenograft models of hematological and solid malignancies^6–8^. The mechanism by which that occurs is not elucidated but studies suggest that cancer cell differentiation^8^ contributes to the process. It is appreciated that differentiation of cells with self-renewal potential increases the sensitivity chemotherapy^37,38^. This correlates with the observation that epigenetic therapies sensitize tumors to chemotherapy treatments^10,39,40^. DNA-demethylating agents also alter the immune response by reactivating cancer testis (CT) antigens^41^, increasing endogenous retroviruses and repetitive elements^7,33^, triggering interferon response thus sensitizing tumors to checkpoint inhibition^6^. In this study, we use low non-cytotoxic doses of a DNA-demethylating agent, Decitabine (DAC), to identify epigenetically activated pathways in bladder cancer.

We observed for the first time that DAC-induced DNA demethylation upregulated *NOTCH1* mRNA in bladder cancer cells suggesting a functional role for DNA methylation in the transcriptional regulation of *NOTCH1*. We examined 4 and 7 CG dinucleotides in the promoter and enhancer regions, respectively, and detected significant DNA demethylation. Hypomethylation in this locus appears to be uniform it is important to note that other sites in these CG-rich regions were not examined due to technical limitations of PCR amplification. The sites that lay beyond the examined region may not be methylated contributes to presence of baseline ICN1 protein expression in the control cells. ICN1 expression despite methylation in NOTCH promoter regions was also demonstrated by Liu et al.^42^. In this study, cells with siRNA against DNMT1 had demethylation of a greater number of CG sites that enhanced transcription and ultimately NOTCH1 protein expression compared to cells with control siRNA^42^. It is likely that passive demethylation of the CG-rich promoter region by DAC treatment, via DNMT1 protein depletion, promotes open chromatin and allows access of transcriptional machinery at *NOTCH1* locus. It is also possible that factors other than DNA methylation contribute to regulation of NOTCH1 expression. For example: We did not detect ICN1 protein in B01 and B02 cells cultured in cholera toxin containing media possibly due to cholera toxin induced degradation^26^. These results suggest that there are additional post-translational mechanisms that govern NOTCH1 expression. Here, we show for the first time that NOTCH1 expression is induced by low non-cytotoxic doses of a DNA-demethylating agent. Importantly, epigenetic regulation of NOTCH1 expression leads to activation of downstream effectors that may possess tumor-suppressive properties in bladder cancer.

NOTCH proteins are structurally conserved with a high degree of homology between NOTCH1 and NOTCH2^43, 44^. Despite the structural similarity, NOTCH1 exerts tumor suppressive whereas NOTCH2 exerts oncogenic actions via different downstream signaling targets in other malignancies^45,46^. In bladder cancer, NOTCH2 increases cell proliferation and invasion by regulating EMT genes *in vitro* and *in vivo* ^47^. NOTCH2 copy number gains are frequent in MIBC patients and correlate with worse overall survival^47^. On the other hand, NOTCH1 is lost and expression of its downstream targets HES-1 and JAGGED-1 are lower in aggressive MIBC^12–14^. The function and potentially compensatory or opposing roles of NOTCH proteins have not yet been well delineated in bladder cancer. For example: studies show that NOTCH1 is oncogenic and increases cell proliferation and invasive potential of bladder cancer cells *in vitro* ^48,49^. These studies used different approaches to reduce NOTCH1 expression namely siRNA^48^ and γ-Secretase inhibitor^49^. γ-Secretase is important for not only cleaving the intracellular domain of NOTCH1 but also cleaving NOTCH2 to activate NOTCH2 signaling^50^. It is possible that using γ-secretase inhibitors will also reduce NOTCH2 activation. The potential off-target effects of siRNAs^51^ and γ-secretase inhibition that affect both NOTCH1 and NOTCH2^48,49^ expression can account for the increased cell proliferation and invasive capacity of bladder cancer cells. In our study, DAC-induced DNA demethylation of the NOTCH1 promoter and enhancer regions upregulated NOTCH1 gene transcription with increase in ICN1 protein, but we are yet to determine the effects of DAC on NOTCH2 signaling.

We also observed for the first time that DAC treatment and/or NOTCH1 mediated IL-6 release resulted in downregulation of CK5 protein expression. Although we attribute the induction of IL-6 mostly to the NOTCH1 signaling we suspect, based on previous studies^31,32^ that increase in double-stranded mRNA sensors also contributes to IL-6 release in DAC-treated cells. Knockout of individual double-stranded RNA sensors could potentially confirm this hypothesis, however other components of this pathway can functionally compensate for depletion of another component. Furthermore, increased IL-6 in DAC-treated B01 and B02 cells cultured in specialized media occurs without NOTCH1 activation. This suggests that double-stranded RNAs can potentially lead to IL-6 release in cells without NOTCH1 activation. IL-6, a pro-inflammatory cytokine, activates the JAK/STAT pathway mostly associated with worse prognosis in cancers^16,17,52^. In bladder cancer, IL-6 through DNMT1 upregulation increases tumorigenic properties of bladder cancer cells *in vitro* and *in vivo* ^53^. In our studies, DNMT1 protein is depleted by DAC through its mechanism of action therefore counteracting IL-6 mediated induction of DNMT1 expression. DAC/NOTCH1 mediated IL-6 release did not maintain increased phospho-STAT3 levels or cause DNA damage. Our results suggest that in the absence of DNA damage, DNMT1, and JAK/STAT signaling, IL-6 release most likely plays a role in differentiation, as observed by reduced CK-5 expression, and elicits anti-tumor activity in MIBC cells. The tumor-suppressive role of IL-6 may be further clarified by the outcome of an ongoing clinical trial combining DAC and Ruxolitinib, a JAK/STAT inhibitor [NCT02076191]. IL-6 is also important in recruiting immune cells into tumor microenviroment^54^. It is possible that DAC and/or NOTCH1 mediated IL-6 release activates paracrine IL-6 signaling in the microenvironment to enhance immune cell infiltration in MIBC tumors.

The first line therapy for patients with locally advanced and metastatic disease has been cisplatin-based chemotherapy (CBC). Patients who fail CBC encounter limited treatment options. Only recently checkpoint based immune therapies have been approved for treating patients with metastatic disease^55^. DNA-demethylating agents such as DAC can be combined with checkpoint based immunotherapy to boost the immune system through increased IFN response^6,7^. This will provide additional therapeutic alternatives to CBC for advanced stage MIBC patients. Another option can be to utilize DAC-induced molecular pathways to potentiate CBC or re-sensitize patients to CBC. NOTCH1 upregulation can increase cisplatin sensitivity based on studies in osteosarcomas^15^ and in our studies we show that NOTCH1 expression can be induced by a DNA-demethylating agent. DAC treatment also lowers CK5 protein expression indicating a reduction in the population of cells with self-renewing capacity. Increased frequency of cells with self-renewal capacity is often associated with cisplatin resistance in bladder cancer cells^56^. Therefore, DAC through multiple pathways may enhance cisplatin activity in MIBC patients with no other therapeutic alternatives. DNA demethylation based epigenetic therapy in combination with CBC or checkpoint based immune therapy is promising and should be explored in treatment of MIBC.

## Materials and methods

### Cell lines, reagents, and treatments

Four bladder cancer cell lines HT1376, T24, BLCAb001^24,25^ (B01), and BLCAb002^24,25^ (B02) were used in this study. B01 and B02 cells were developed in the Pili lab^24,25^. HT1376 cells (ATCC,CRL-1472) were cultured in MEM (Corning, 10–010CV) with Non-essential amino acids, T24 cells (ATCC, HTB-4) were cultured in McCoy’s (Corning, 10–050CV) and B01 and B02 cells were cultured as specified in previous studies^24,25^. Media was supplemented with 10% fetal bovine serum, and Penicillin/Streptomycin and cells were incubated at 37 °C and 5% CO_2_ concentration. All the cell lines were tested for mycoplasma (Lonza, LT07-218) once every 2 months. 1 mM stock concentrations of Decitabine (Sigma, A3656) was solubilized in aqueous solution and stored at −20 °C for further use. Decitabine solutions were not thawed more than once, and 0.1 and 1 µM DAC solutions for cell treatments were made in media for each cell line. ICN1 plasmid from Addgene (17,623) was transiently transfected into cell lines using Lipofectamine 3000 (ThermoFisher Scientific, L3000008) and collected at 72 h for further analysis. IL-6 (Affymetrix, 14-8069-80) was prepared according to manufacturer’s recommendation and used at 50 ng/ml for up to 7 days. Monoclonal antibody against IL-6 (Affymetrix, 16-7069-85) was used at a concentration of 1 ng/ml for rescue experiments in DAC-treated cells.

### Western blots and qPCR

Cells were plated in 6 well plates and treated with 0.1 and 1 µM DAC every 48 h for up to 5 days. Cells were collected using RIPA buffer with protease and phosphatase inhibitors. Lysates were spun at 16,000 × *g* for 10 min at 4 °C, and protein concentration in the resulting supernatant was measured by Bio-Rad DC protein assay (Bio-Rad, 5000116). 40 µg of total cell lysate was loaded on Bio-Rad 4–15% gradient gels (567–1084) and was transferred onto a nitrocellulose membrane followed by blocking with 5% BSA (Sigma, A9647). The following primary and secondary antibodies were used in our studies: DNMT1 (Cell Signaling, 5119), NOTCH1 (Cell Signaling), p-STAT3 (Cell Signaling, 9145), STAT3 (Cell Signaling, 4904), CK5 (Covance, PRB-160P), Secondary Rabbit (GE Biosciences NA934), and Secondary mouse (GE Biosciences NA931V) at recommended dilutions. mRNA was isolated using a kit (ZymoResearch, R2052) followed by cDNA synthesis (Bio-Rad, 170-8891). qPCR was carried out using SYBR green (Bio-Rad, 172-5121) and primers for the genes of interest are listed below. ΔΔCt values were used to calculate fold changes of DAC-treated cells over control cells

### Illumina Infinium 450 K Bead Chip Assay, pyrosequencing, and Paired-end RNA sequencing

174 bladder tumor samples (including 74 matched non-tumor and tumors) obtained from patients at Roswell Park Cancer Institute were analyzed for methylome changes with the 450 K array. BeadScan in the GenomeStudio module was used to extract intensities, summarized by BeadStudio and processed by R. Design bias was corrected by Swan normalization and loci with median detection *p*-value <0.05 were further analyzed. β-value change of 0.17 was set as the threshold for establishing the difference between tumor and non-tumor samples. Pathway enrichment program in Genego was used to identify the top 10 pathways that contained DAC-mediated hypomethylated CGs. Network enrichment program in Genego was similarly used for identifying top 10 gene networks that are hypermethylated in bladder tumors. For pyrosequencing, DNA was isolated (Qiagen, 51304) and bisulfite converted (ZymoResearch, D5031). The bisulfite converted products were amplified using PCR primers targeting specific genes (listed below) and run on an agarose gel to check for methylation bias. Pyromark Reagents (Qiagen, 972804), Pyromark Cartridge (Qiagen, 979004) and Pyromark plates (Qiagen, 979002) were used for running samples on the pyrosequencer. The PCR products were immobilized on Sepharose beads (GE Healthcare, 17-5113-01), captured using vacuum, washed with 70% ethanol, denaturation solution (0.2 M NaOH) and washing buffer (10 mM Tris-Acetate, pH 7.6). Beads were released in annealing buffer and heated for 1 min at 80 °C and run on PyroMark Q24 that analyzed percent methylation at each CG site. 1.5 µg of RNA was used for Paired-end RNA sequencing, and fastQC was used to check the base quality of the raw reads. Reads were aligned with Tophat 2 to human genome b37 from Ensembl. Raw read counts were analyzed using R, and differentially expressed genes between control and DAC-treated cells were identified. Similar RNA sequencing analysis was performed for 66 bladder tumor samples (including 15 matched tumor and non-tumor samples). These were a subset of the samples that were analyzed for changes in DNA methylation by the 450 K array. Pathway enrichment program in Genego, available through Metacore, was used to identify the top 10 pathways affected by DAC treatment and altered in bladder tumor samples. qPCR was used to validate the genes identified by Genego in the pathway enrichment analysis.

### β-gal assay and IL-6 ELISA

β-gal positivity was visualized using a colorimetric kit (Biovision, K320-250). Briefly, cells were washed twice with PBS, fixed with fixing solution for 10 min, washed twice with PBS and stained with 2 mg/ml of X-gal overnight at 37 °C. The next day X-gal solution was replaced with 70% glycerol and images were taken under bright field microscopy. For IL-6 ELISA, cells were collected by trypsin, and cell count and viability were measured on a Vi-Cell automated counter. The cell supernatants were stored at −80 °C for IL-6 ELISA. IL-6 release in the supernatants was measured (Affymetrix eBioscience, 88-7066-22). IL-6 concentrations from absorbance measurements were normalized to cell count, and pg/mL per 10^6^ cells were used to analyze the difference between DAC-treated or ICN1 overexpression and control cells.

### Cell counts, cell viability, and clonogenics

Cells were collected by trypsin and cell counts, and cell viability were obtained by an automated Vi-cell counter using trypan blue exclusion method. For clonogenics, cells were collected on day 3 of DAC treatment, and 50–100 cells were resuspended (depending on the cell line) and plated in a 12-well plate. Media was refreshed every 3 days, and the clones were collected on day 10 after plating. The clones were washed and stained with crystal violet containing 20% methanol for an hour followed by washing with distilled water and dried overnight. The numbers of clones were manually counted, and DAC treatment was compared to control cells.

### Flow cytometry and cell size analysis

Cells were collected by trypsin on day 5 of DAC treatment, washed twice with PBS and fixed in 70% ethanol overnight. Fixed cells were washed twice with PBS, stained with propidium iodide containing RNase I and were run on BD Fortessa for cell cycle analysis. FCS express and forward scatter-width, known to provide an accurate assessment of cell size,^57^ was used to calculate relative cell size differences between control and DAC-treated cells.

### Patient data

MIBC patients from Roswell Park Cancer Institute were collected between the years 1995–2011. Methylation array (*n* = 174) and RNA sequencing analysis (*n* = 66) were carried out as described before. The TCGA bladder sample data set for RNA-seq and clinical data were downloaded (October 2016) and analyzed for the genes of our interest. There were 412 samples for which RNA-seq, p53 mutations, and metastatic disease was available and 120 samples for which chemotherapy data was available. RNA-seq data were normalized using *z*-scores, and unsupervised hierarchical clustering was performed to evaluate correlations between transcript levels, p53 mutations, metastatic disease and chemotherapy response. A *z*-score threshold of +1.5 was set for NOTCH1 and IL-6 up and downregulation in patient samples. cBioportal was further used to observe survival differences in genes of our interest^58,59^. For heatmaps in Fig. 4 column *z*-scores were calculated to compare the levels of gene expression within the same tumor. The patient demographic for the RPCI data set is presented in Supplementary Table 3. The patient demographic for the TCGA data set has been previously published^60^.

### Statistical analysis

GraphPad Prism software was used for plotting graphs and statistical analysis. Student’s *t*-test was used to compare DAC treatments with control cells. The time-to-event outcomes in the RPCI cohort were reported by NOTCH1 status using standard Kaplan-Meier methods; with comparisons made using the log-rank test. All analyses of MIBC patients in the Roswell Park cohort were conducted in SAS v9.4 (Cary, NC) at a significance level of 0.05.

## Electronic supplementary material

Supplementary figure and table legends

Supplementary Figure 1

Supplementary Figure 2

Supplementary Figure 3

Supplementary Figure 4

Supplementary Figure 5

Supplementary Table 1

Supplementary Table 2

Supplementary Table 3
