# Supplementary material for: Decitabine, a DNA-demethylating agent, promotes differentiation via NOTCH1 signaling and alters immune-related pathways in muscle-invasive bladder cancer
Source: Cell Death Dis. 2017 Dec 14;8(12):3217. doi: 10.1038/s41419-017-0024-5 (PMC5870579; doi:10.1038/s41419-017-0024-5)
Supplement: Supplementary file 1 — Supplementary figure and table legends [file 41419_2017_24_MOESM1_ESM.docx]

**Supplementary figures and tables**

**Supplementary figure 1. DAC reduces cell proliferation without causing toxicity and alters cell morphology in bladder tumor cells *in vitro***. Bladder tumor cells were treated with DAC every 48 hours for up to 5 days. **a.** DAC treatment lead to sustained depletion of DNMT1 protein with GAPDH used as a loading control. The image is a representative from one of the triplicate experiments. **b.** On Day 3 of DAC treatment, a decrease in LINE-1 methylation was observed in the cell lines. The Y-axis represents percent methylation in control, and DAC-treated cells with error bars indicating standard error of means from technical and biological duplicates. **c.** 0.1µM and 1µM DAC did not reduce viability by more than 20% in bladder tumor cells. The Y-axis represents percentage changes in viability in DAC-treated cells as compared to control cells. Error bars indicate standard error of means from triplicate experiments and technical duplicates in each experiment. **d.** On Day 5 of DAC treatment, cells analyzed were analyzed for relative cell size change by flow cytometry. The Y-axis represents percentage changes in cell size in DAC-treated cells as compared to control cells. Error bars indicate standard error of means from triplicate experiments and technical duplicates in each experiment. **e.** Representative images of forward scatter analysis from all four cell lines are presented. Black curve indicates control cells, red curve indicates 0.1µM DAC treated cells and blue curve indicates 1µM DAC treated cells. *p<0.05, **p<0.01, #p=0.054, ^p=0.056.

**Supplementary figure 2. NOTCH1 pathway enriched in RNA-sequencing data correlates with better outcomes in bladder cancer patients. a.** RNA sequencing heatmap of T24 cells at 72 hours with an additional treatment at 48 hours showed upregulation of RNA transcripts by DAC. Red indicates higher gene expression values and green indicates lower gene expression. Log fold change greater than two was considered to be significantly (p<0.05) upregulated in DAC-treated cells as compared to control cells. **b.** NOTCH1 mRNA levels were upregulated in 12% of Roswell Park patients. Patients with increased NOTCH1 transcript levels trended towards better overall and progression-free survival as compared to patients with who did not exhibit *NOTCH1* upregulation. **c.** mRNA and protein overexpression was found in 12% of TCGA bladder tumors (Total n=412). Overall and progression-free survival of patients with NOTCH1 overexpression trended towards increased survival in months as compared to patients with no alterations. **d.** TCGA dataset revealed that increased overall methylation at NOTCH1 leads to reduced mRNA expression in a subset of tumors.

**Supplementary figure 3. Transient ICN1 overexpression reduces cell proliferation and changes cell morphology without increasing β-gal and p16 expression.** **a.** Transient ICN1 overexpression reduced cell proliferation measured at 72 hours compared to control cells. The Y-axis represents percentage changes in cell counts in ICN1 overexpressing cells as compared to control cells. Error bars are standard error of means from triplicate experiments and technical duplicates in each experiment. **b.** Bright field images of Giemsa staining showed morphological changes in ICN1 overexpressing cells. ICN1 overexpressing cells appeared enlarged and flattened as compared to control cells. The images are representative of duplicate experiments. Students t-test was used to compare treated and control cells. *p<0.05, **p<0.01. **c and e.** β-gal positivity occurred in a fraction of DAC-treated and ICN1 overexpressing tumor cells. The images are representative of triplicate experiments performed. **_______** represents 100µm in brightfield images. **d and f.** p16 protein expression measured by Western blot did not increase by DAC treatment and ICN1 overexpression. GAPDH and Tubulin were used as loading controls. The Western blot image is a representative of triplicate experiments. **g.** DAC treatment increased p27 protein expression measured by Western blot in a subset of cell lines. GAPDH was used as loading controls. The Western blot image is a representative of triplicate experiments.

**Supplementary figure 4. DAC induces mRNA levels of IFN-γ pathway by increasing levels of viral infection response genes. a-d** Bladder tumor cell lines were treated with DAC every 48 hours for up to 5 days. mRNA levels of *IRF7*, *IFN-β* and downstream signals *IFI27* and *IFI44* increases in DAC-treated cells. The Y-axis indicates average fold change over control cells from triplicate experiments and technical duplicates with error bars indicating standard error of means. Student’s t-test was used to compare control and treated cells. **e and f.** Long exposure (10 minutes) of γH2A.X protein expression showed minimal induction after DAC treatment. Total histone H3 was used as loading control, and the image is a representative from one of the triplicate experiments. **g.** DAC treatment significantly reduced cell proliferation in HT1376 and B02 cells. 50ng/ml IL-6 addition did not significantly change cell proliferation in both cell lines. Monoclonal antibody against IL-6 increased cell proliferation only in B02 cells. The Y-axis represents percentage changes in cell counts in treated cells as compared to control cells. Error bars indicate standard error of means from triplicate experiments and technical duplicates in each experiment. *p<0.05, **p<0.01.

**Supplementary figure 5. DAC does not maintain sustained levels of p-STAT3 protein expression.** Bladder tumor cells were treated with DAC every 48 hours for up to 5 days. **a.** p-STAT3 and STAT3 protein levels increased in DAC treatment in a subset of bladder tumor cell lines. p-STAT3 and STAT3 were measured 30 minutes after DAC addition on day 4. GAPDH was used as the loading control. The western blot image is a representative of triplicate experiments. **b.** Cells harvested on Day 5 of DAC treatment did not express p-STAT3 and STAT3. **c.** IL-6 addition for 30 minutes increased p-STAT3 levels in all four cell lines. GAPDH was used as the loading control. The western blot image is a representative of triplicate experiments.

**Supplementary Table 1** shows gene set enrichment analysis of RNA transcripts upregulated in DAC-treated T24 cells

**Supplementary Table 2** represents patient demographics of the Roswell Park Cohort

**Supplementary Table 3** represents the hypermethylated genes in the Roswell Park Cohort
