# Supplementary figures and images for: Decitabine, a DNA-demethylating agent, promotes differentiation via NOTCH1 signaling and alters immune-related pathways in muscle-invasive bladder cancer
Source: Cell Death Dis. 2017 Dec 14;8(12):3217. doi: 10.1038/s41419-017-0024-5 (PMC5870579; doi:10.1038/s41419-017-0024-5)

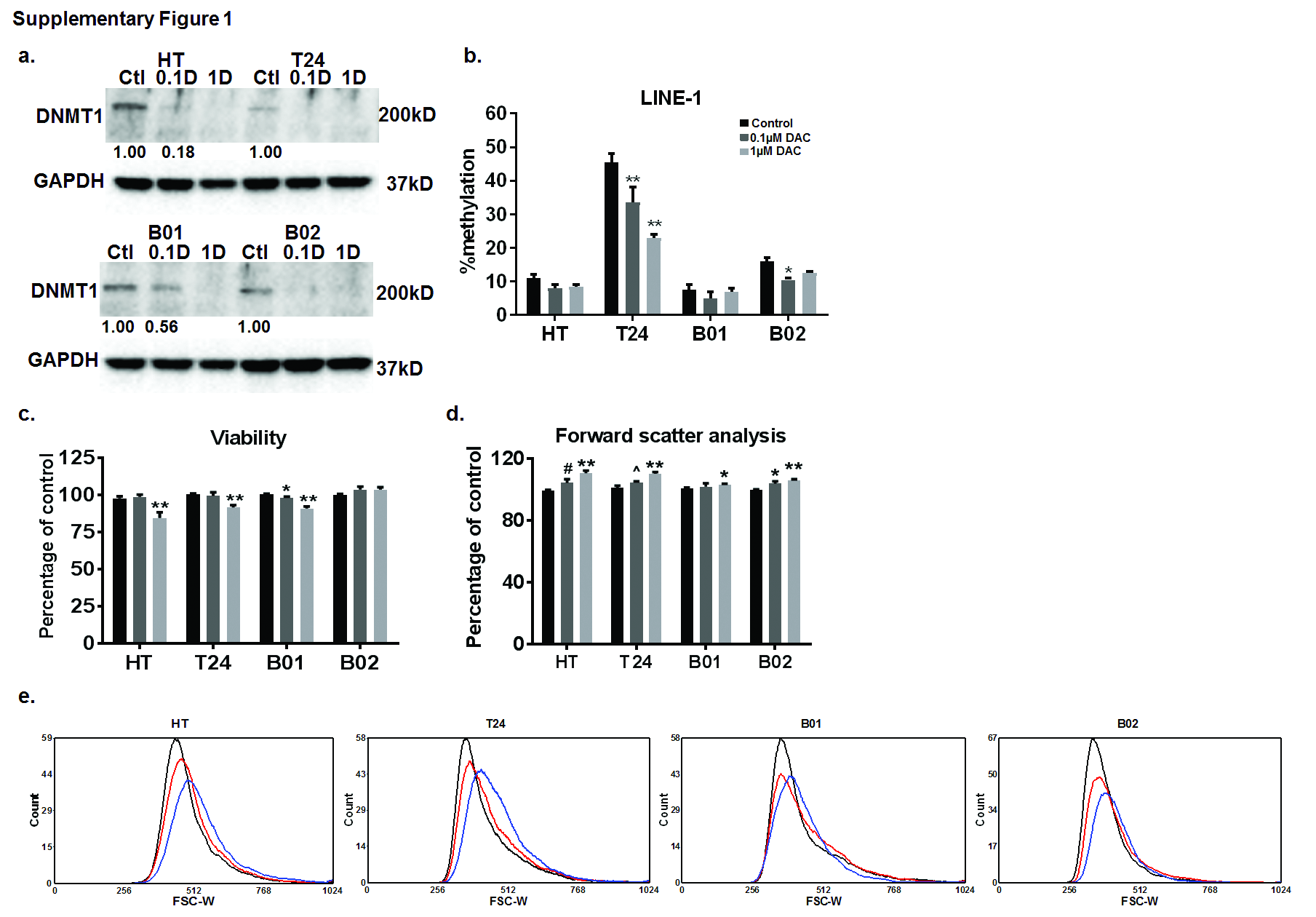

Supplement: Supplementary file 2 — Supplementary Figure 1 [file 41419_2017_24_MOESM2_ESM.tif]

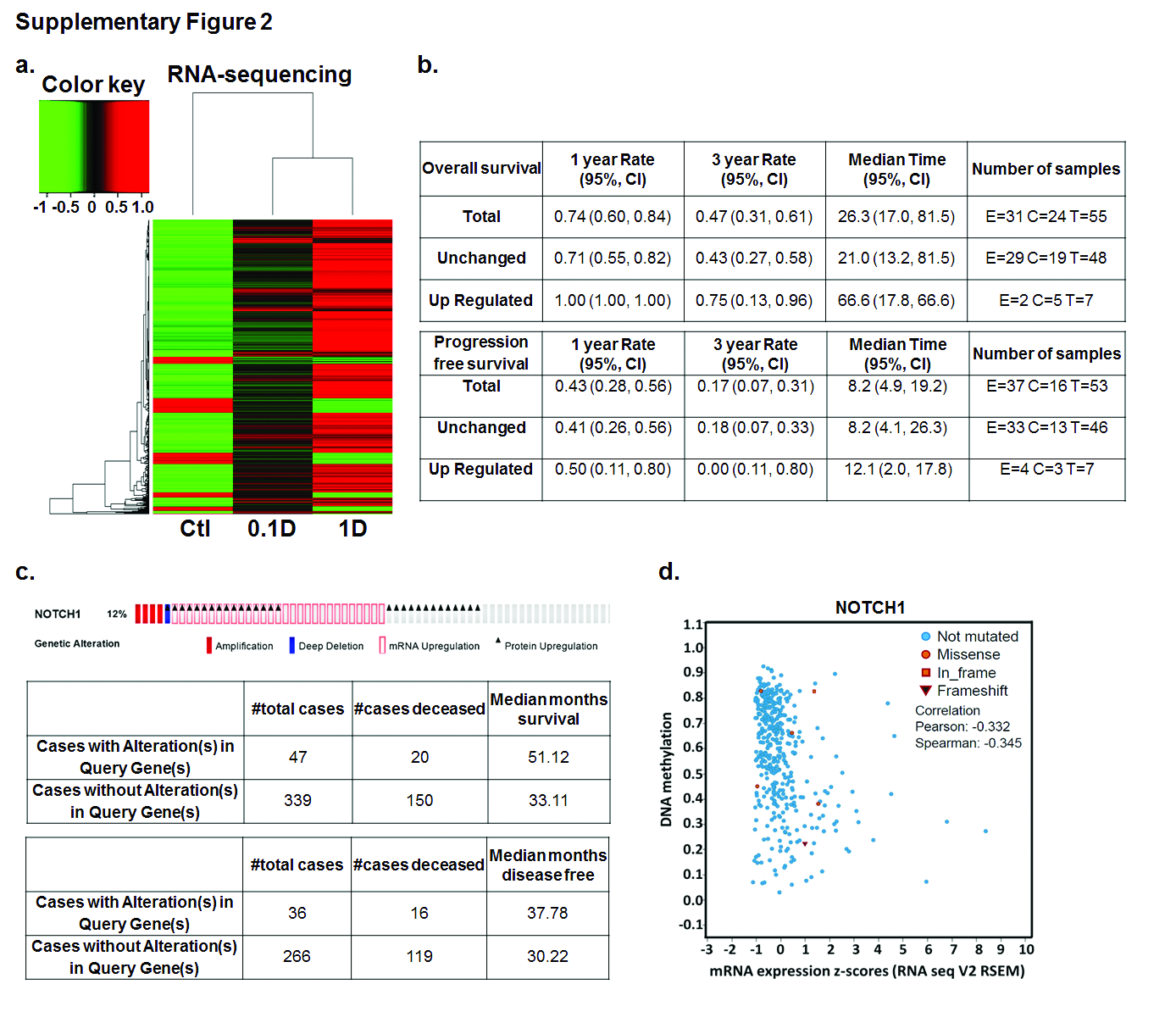

Supplement: Supplementary file 3 — Supplementary Figure 2 [file 41419_2017_24_MOESM3_ESM.tif]

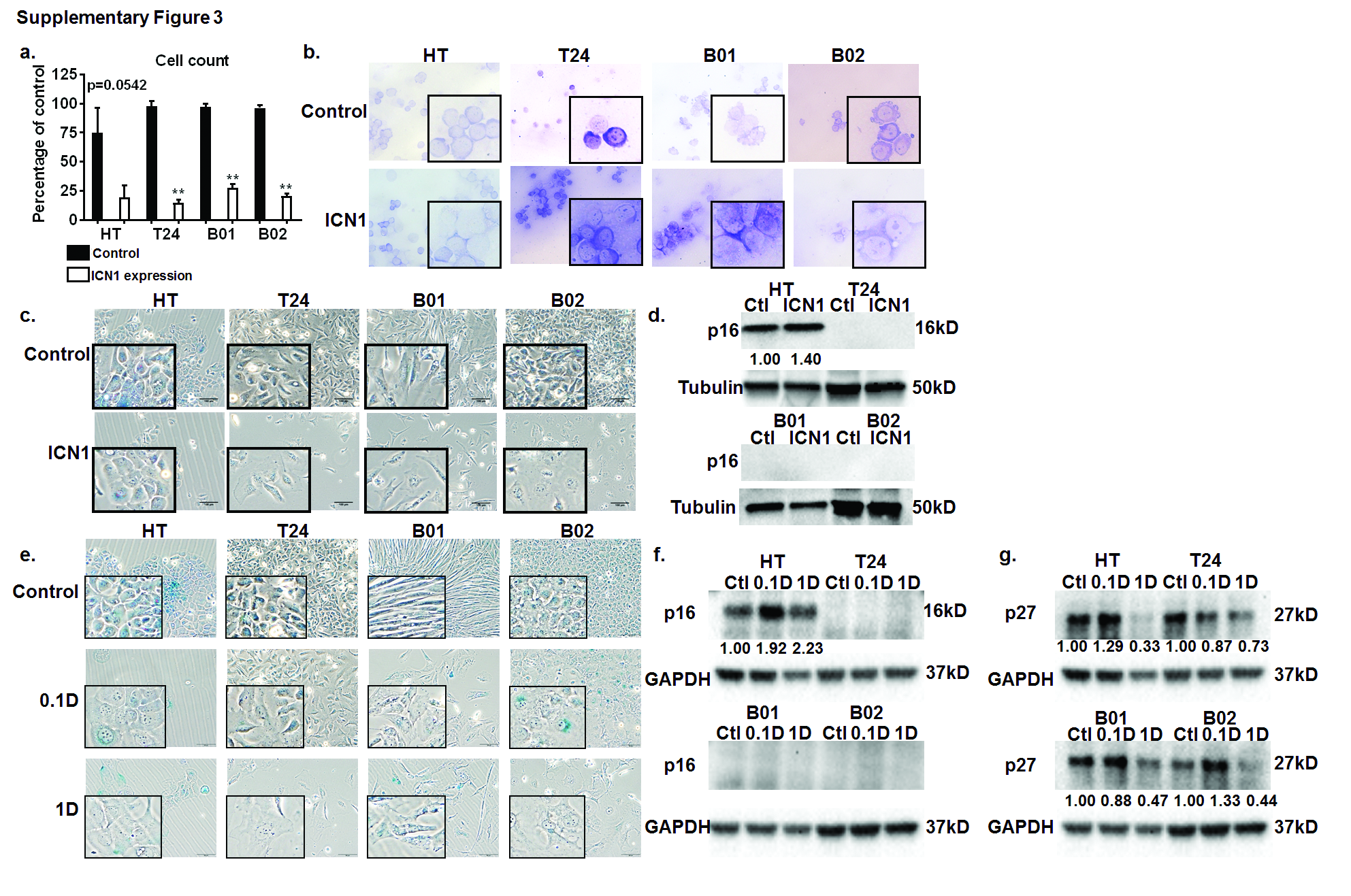

Supplement: Supplementary file 4 — Supplementary Figure 3 [file 41419_2017_24_MOESM4_ESM.tif]

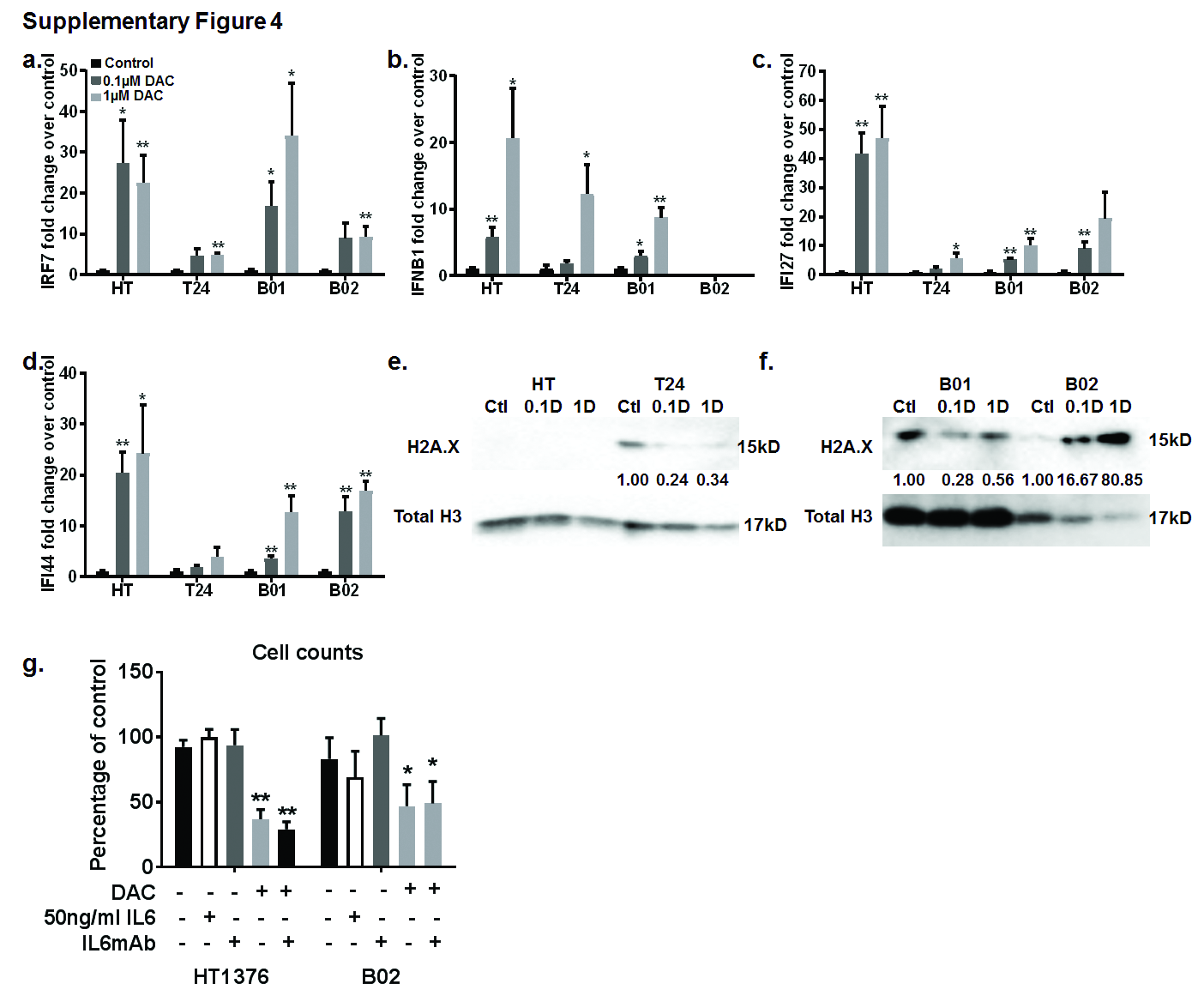

Supplement: Supplementary file 5 — Supplementary Figure 4 [file 41419_2017_24_MOESM5_ESM.tif]

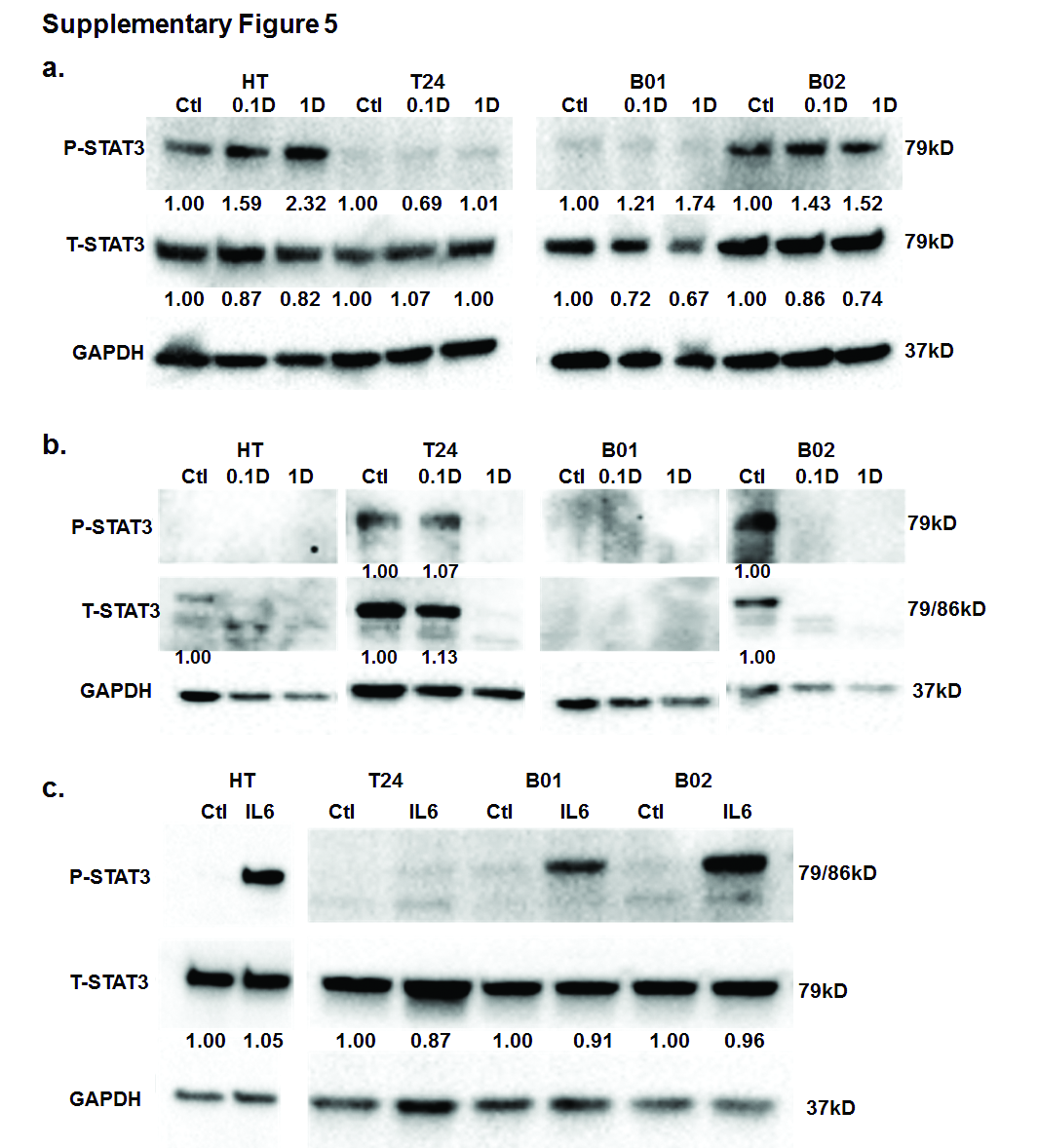

Supplement: Supplementary file 6 — Supplementary Figure 5 [file 41419_2017_24_MOESM6_ESM.tif]
